# Case Report: Temporary pacing using active fixation lead and invasive electrophysiology studies for immune checkpoint inhibitor associated reversible advanced atrioventricular block

**DOI:** 10.3389/fcvm.2024.1336609

**Published:** 2024-02-05

**Authors:** Yifan Wang, Min Qian, Xiaofeng Jin, Jiaqi Wang, Taibo Chen, Peng Gao, Zhongwei Cheng, Jinzhi Lai, Yongtai Liu, Jingbo Fan, Lihua Zhang, Kangan Cheng, Hua Deng, Quan Fang, Deyan Yang

**Affiliations:** ^1^ Medical Intensive Care Unit, State Key Laboratory of Complex Severe and Rare Diseases, Peking Union Medical College Hospital, Peking Union Medical College, Chinese Academy of Medical Sciences, Beijing, China; ^2^ Department of Neurology, Peking Union Medical College Hospital, Peking Union Medical College and Chinese Academy of Medical Sciences, Beijing, China; ^3^ Department of Cardiology, Peking Union Medical College Hospital, Peking Union Medical College and Chinese Academy of Medical Sciences, Beijing, China

**Keywords:** immune checkpoint inhibitors, invasive electrophysiology studies, temporary pacing, advanced atrioventricular block, case report

## Abstract

A case of immune checkpoint inhibitors (ICIs)-associated myocarditis with reversible advanced atrioventricular block (AVB) was reported. We innovatively used active fixation lead connected to an external device for prolonged temporary pacing until atrioventricular conduction recovered. Invasive electrophysiology studies were performed to evaluate atrioventricular conduction in detail. Long-term follow-up for nearly 120-days and repeated long-term electrocardiography was conducted to ensure the conduction system was truly recovered.

## 1 Introduction

The use of immune checkpoint inhibitors (ICIs) greatly improves the clinical outcomes of patients with cancer. However, the application of ICIs may lead to immune-related adverse events (irAEs). Myocarditis is the most commonly considered cardiovascular irAEs. Previous studies have reviewed different databases and found the incidence of ICIs-associated myocarditis is up to 1% and the fatality is over 50% (1). Complete heart block developed in 17.0% of ICIs-associated myocarditis and was related to higher all-cause mortality (2).

Permanent pacing is indicated in patients with complete atrioventricular block (AVB) or advanced AVB (3). Several previous cases have reported the possibility to recover from ICIs-associated complete AVB, and the duration from heart block onset to recovery was highly various from days to months (4–7). On one hand, temporary transvenous passive pacing lead is unstable and might cause procedure-related complications such as electrode displacement, thrombotic events and immobilization when left in place for long duration (8–10). On the other hand, permanent pacemaker should not be implanted in patient with reversible heart block.

A percutaneous transvenous active fixation lead connected to an external device is safe and comfortable for patients requiring prolonged temporary pacing and has been used as bridging therapy in patients who have underwent cardiac implantable electronic device (CIED) extraction for infection and require prolonged antibiotic treatment (3, 11). The role of pacing with percutaneous transvenous active fixation lead has not been fully studied in ICIs-associated heart block.

We reported a case of ICIs-associated myocarditis complicated with advanced AVB. Considering the possibility of AVB recovery, an externalized active fixation temporary pacing lead was placed and invasive electrophysiology study (EPS) was performed. After 3 months, long-term electrocardiography (ECG) monitoring confirmed the recovery of advanced AVB and the active fixation lead and the externalized pacemaker was removed. The patient was stable during follow-up.

## 2 Case description

A 60-year-old man with hypertension and no previous cardiac disease presented to our emergency department with shortness of breath, myalgias of limbs for 5 days and diplopia for 2 days. He had a 5-year history of poorly to moderately-differentiated adenocarcinoma of left lung and developed brain metastases. He underwent pulmonary lobectomy. Although receiving standard chemotherapy, targeted therapy and radiotherapy, he had intracranial progression for brain metastases.

One cycle of pembrolizumab (200 mg) combined with axitinib and anlotinib were administered. His baseline ECG before pembrolizumab initiation showed a normal sinus rhythm without bundle branch block and atrioventricular block. Fifteen days after receiving his first infusion of pembrolizumab, the patient experienced shortness of breath during exertion and myalgias of limbs and fatigue. His symptoms deteriorated and began to have diplopia 3 days later. His ECG in local hospital showed sinus tachycardias with new onset first-degree AVB and complete right bundle branch block (CRBBB). No specific treatment was given and his symptoms aggravated. He was transferred to our emergency department (ED).

On initial evaluation, his heart rate (HR) was 130 beats/min with blood pressure of 129/90 mmHg, SpO_2_ was 96% at room air and respiratory rate was 16 breaths/min. Physical examination revealed blepharoptosis and decreased muscle strength of the extremities. Cardiac auscultation revealed normal heart sound without murmurs. Levels of high-sensitive C-reactive protein (hsCRP), serum high-sensitive cardiac troponin I (hscTnI), creatine kinase (CK), and creatinine kinase-myocardial band (CK-MB) were 66 mg/L, 39,778 ng/L, 18,325 U/L and 163.6 ug/L, respectively. N-terminal pro-B-type natriuretic peptide (NT-proBNP) was 2,293 pg/ml.

**Figure 1:**
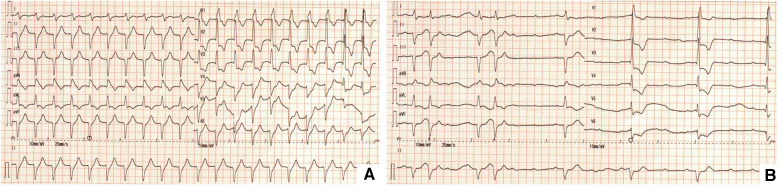
(**A**) Ventricular tachycardia. (**B**) Advanced AVB.

His initial ECG in ED showed ventricular tachycardia with heart rate at 130 beats/min (Figure 1A). During his stay in ED, syncope occurred recurrently. Rhythm monitoring and repeat ECG showed advanced AVB with ventricular escape and the HR was 20–50 beats/min during syncope (Figure 1B).

Because the administration of atropine and intravenous isoproterenol did not stabilize his heart rate, a temporary pacing lead was emergently placed via right femoral vein. The patient was then stable and transthoracic echocardiogram (TTE) was performed which showed a normal left ventricular systolic function (left ventricular ejection fraction of 66%) without regional wall motion abnormality.

According to his medical history, symptoms and laboratory results, ICIs-associated myositis complicated with advanced AV block and heart failure with preserved ejection fraction (HFpEF) were diagnosed. Giving the presence of hemodynamic instability, high intensity treatment of intravenous methylprednisolone of 1 g/day (Then, sequential 80 mg/day intravenous methylprednisolone was given for 7 days, 40 mg/days intravenous methylprednisolone for 2 weeks. After that, corticosteroids were taken orally and gradually tapered from 36 mg/days by 4 mg/week lasting 2 months.) and intravenous immunoglobulin (IVIG) of 20 g/day were given for 3 days. Because no significant improvement had been seen after pulse corticosteroid dosing within 24 h, tocilizumab injection of 640 mg was used for 1 day following the recommendation of guidelines (12, 13). Furosemide was given for diuresis.

**Figure 2:**
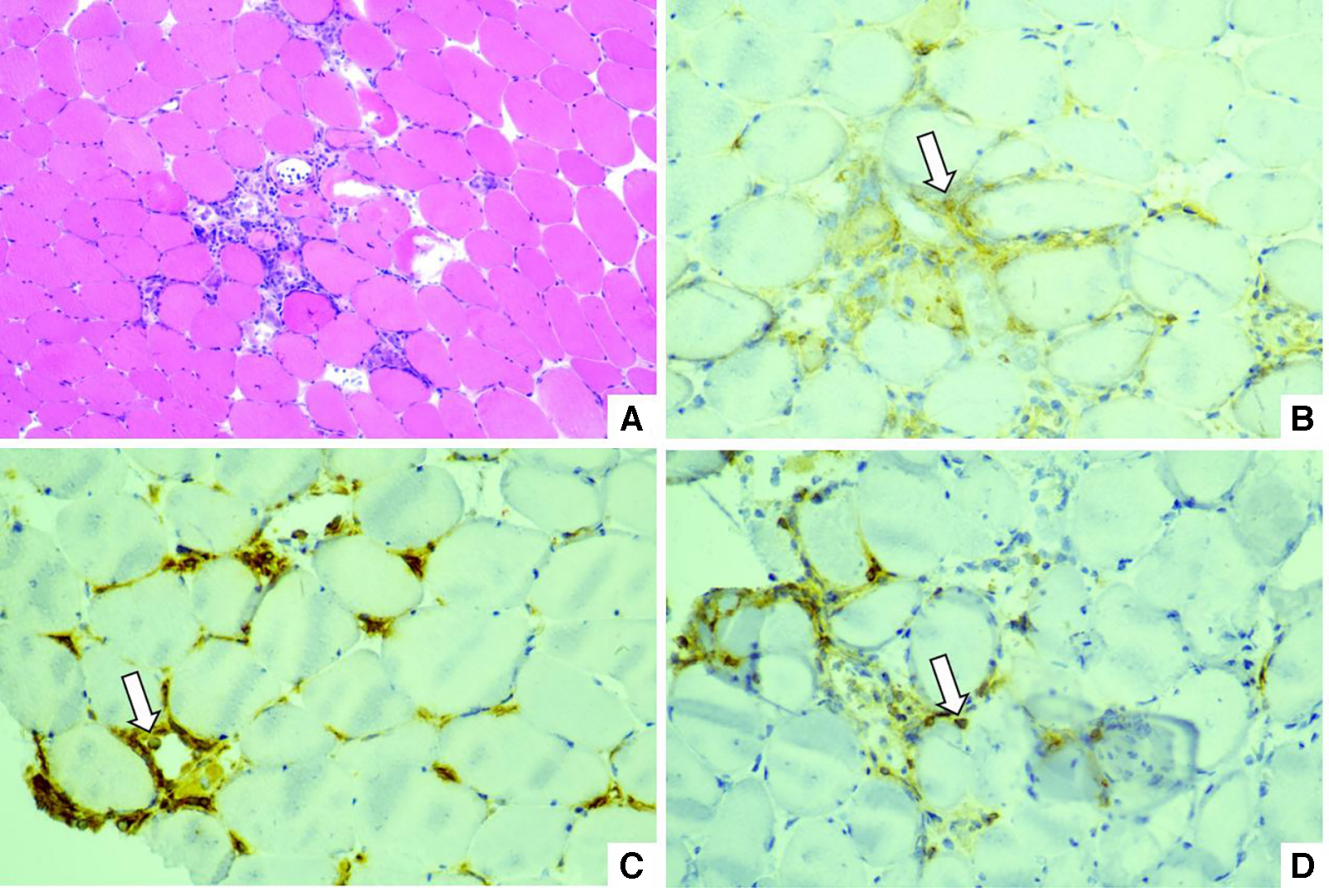
(**A**) Hematoxylin-eosin staining: infiltration of mononuclear cells between myocytes. (**B**) Immunohistochemical staining of CD4: white arrow. (**C**) Immunohistochemical staining of CD68: white arrow. (**D**) Immunohistochemical staining of CD20: white arrow.

After 3 days of medical therapy, advanced AVB did not recovered and an active fixation lead (Medtronic 5076-58) connected with an externalized generator (Medtronic Adapta ADSR01) was placed via right internal jugular vein for temporary pacing for the possibility of AVB recovery. The lower rate was set at 80 beats/min to inhibit ventricular tachycardia. Muscle biopsy was performed and showed infiltration of CD4+, CD20+ and CD68+ cells in necrosis areas with up-regulation of major histocompatibility complex antigens (MHC) class I in myolemma which indicated an immune-induced myositis (Figure 2). Given the risk of pacing lead displacement during the procedure, endomyocardial biopsy was not performed.

**Figure 3:**
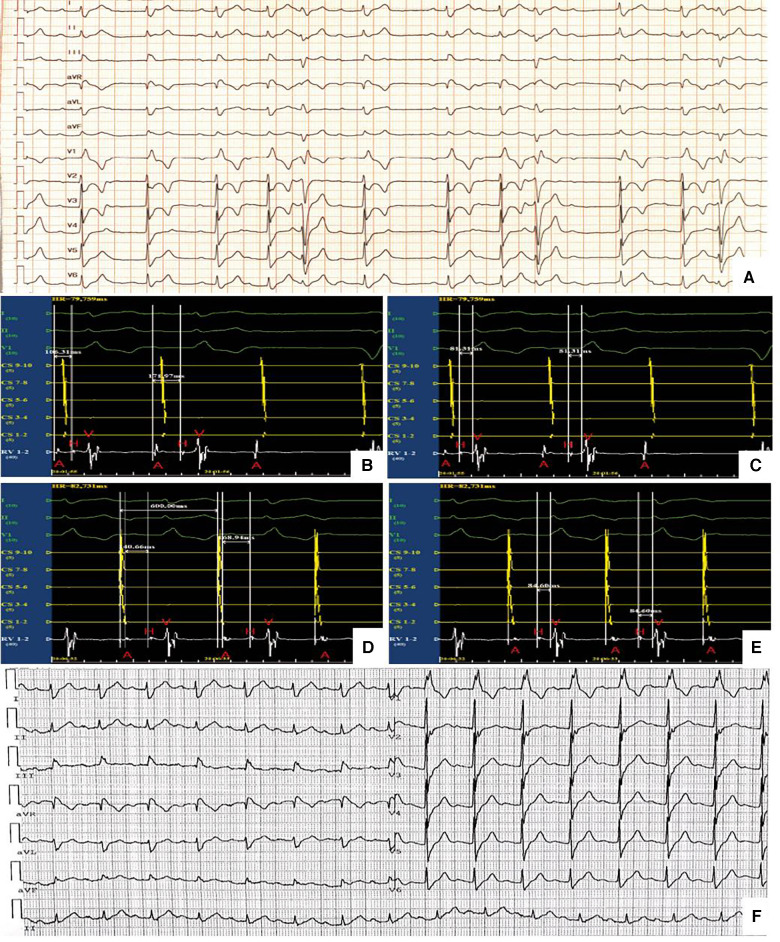
(**A**) Second-degree type I AVB (**B**) and (**C**) EPS at baseline. A, atrial potential; CS, coronary sinus; H, his bundle potential; RV, right ventricle; V, ventricular potential (**D**) and (**E**) EPS during RA pacing. A, atrial potential; CS, coronary sinus; H, his bundle potential; RV, right ventricle; V, ventricular potential. (**F**) First-degree AVB and CRBBB.

After 3 weeks of medical therapy, the patient's symptoms improved significantly and no syncope occurred. Myocardial enzymes continuously decreased to 714 ng/L, 1,009 U/L and 133.1 ug/L for hscTnI, CK and CK-MB respectively. NT-proBNP decreased to 1,770 pg/ml. Repeat ECG and 24 h-holter showed AVB improved from advanced AVB to second-degree type I AVB (Figure 3A) 1 month after implantation of the active fixation lead and externalized generator. Invasive EPS was conducted to demonstrated the conductive abilities. During EPS, a coronary sinus catheter was placed via femoral vein to record atrial potential and a His bundle catheter was placed to record atrial potential, His potential and right ventricular potential. EPS at baseline showed a surface ECG rhythm of second-degree type I AVB with sinus rhythm and 3:2 AV conduction and AH interval was prolonged from 106 ms to 172 ms with a consistent HV interval of 81 ms (Figures 3B,C). During RA pacing at a cycle length of 600 ms, AH interval was prolonged from 141 ms to 169 ms with an unchanged HV interval of 85 ms (Figures 3D,E). The results of EPS demonstrated the heart block of supra-Hisian origin. The patient was discharged home and received periodic follow-up in outpatient department.

Another 5 weeks later, the corticosteroids were stopped and 7-days ECG monitoring showed that average HR was 80 beats/min and maximum HR was 154 beats/min at daytime with 1:1 atrioventricular conduction. Second-degree type I AVB and 2:1 AVB also occurred during sleep. The patient was hospitalized again preparing to remove of pacemaker. The interrogation of the pacemaker confirmed the proportion of ventricular pacing for the last month was only 0.5% and pacing threshold (0.625 mV with pulse width at 0.4 ms), ventricular sensing (15.68 mV–22.40 mV) and the lead impedance (568 *Ω*) were all within normal range. The active fixation lead and externalized generator were then removed 98 days after the first advanced AVB occurrence.

During follow-up and close observation in outpatient for the next 3 weeks, when shortness of breath, myalgias of limbs and diplopia recovered completely, a repeat ECG revealed HR was 94 beats/min and first-degree AVB (PR interval of 234 ms) and CRBBB (Figure 3F). Computed tomography angiography demonstrated moderate narrowing of the left anterior descending coronary artery (LAD). Cardiac MRI was normal without late gadolinium enhancement. A repeat 7-days ECG monitoring still indicated second-degree type I AVB and 2:1 AVB during sleep.

## 3 Discussion

ICIs-associated myocarditis is uncommon and usually severe even fatal especially complicated with AVB. Seventeen percent of ICIs-associated myocarditis complicated with complete heart block in one retrospective study (2). Those patients were more likely to experience all-cause mortality within 30 days of admission (48% vs. 22%) (2). Here, we presented an ICIs-associated myocarditis case with advanced AVB causing recurrent syncope and externalized active fixation lead temporary pacing, instead of permanent pacemaker, was used until AVB restored to second-degree type I AVB which was confirmed by invasive EPS.

Exact mechanisms of conduction disease caused by ICIs is unclear. T cells, antibody and cytokine responses may play a role (14). Reversibility of the conduction abnormality has been reported in previous cases (4). After treatment, the time needed for conduction system to recover varied from 2 days to 2 months. Timely treatment, especially use of corticosteroids may contribute to better clinical outcomes. This may because corticosteroids can suppress lymphocyte activity and inhibit cytokine synthesis (6), thus controlling inflammation of myocardia.

Current guidelines recommended pacing therapy for serious AVB (3, 13).One pooled analysis showed that timely pacemaker implantation is associated with better prognosis in patients with ICIs-related complete AVB (6). Since the uncertainty of the recovery of ICIs-associated conduction abnormalities and the great variability of recovery time, AVB might take several months to restore, so externalized active fixation lead temporary pacing is worth trying. In our case, the patient showed a partially recovery of the cardiac conduction system after 1 month of treatment, implantation of a permanent pacemaker was thus avoided. After the removal of pacemaker, the patient was in good status without syncope.

Invasive EPS was reported in one case to reveal conduction system dysfunction after cemiplimab therapy (15). In that patient, EPS at baseline demonstrated a surface ECG rhythm of high-grade AV block with sinus rhythm with 3:1 to 2:1 AV block with a PR interval and RR interval of 302 ms and 1,350 ms, respectively. AH interval was 108  ms and HV interval was up to 219 ms with 2:1 AV block of infra-hisian nature. The EPS results demonstrated intermittent complete heart block of infra-hisian origin, so permanent pacemaker was implanted. Different from the previous case, EPS of our patient showed a gradually prolongation of AH interval with consistent HV interval demonstrating a supra-hisian block, which may be an indicator for the further recovery of ICIs-associated AV block.

In conclusion, our case emphasizes that advanced AVB due to ICI-associated myocarditis might restore and externalized active fixation lead for prolonged temporary pacing was a reasonable bridging therapy before the implantation of permanent pacemaker. Further studies are warrant to further verified the role of externalized active fixation lead temporary pacing and prognostic values of invasive EPS in treating ICIs-associated conduction abnormalities.

## Data Availability

The original contributions presented in the study are included in the article/**Supplementary Material**, further inquiries can be directed to the corresponding author.
