# Supplementary material for: Case Report: Temporary pacing using active fixation lead and invasive electrophysiology studies for immune checkpoint inhibitor associated reversible advanced atrioventricular block
Source: Front Cardiovasc Med. 2024 Feb 5;11:1336609. doi: 10.3389/fcvm.2024.1336609 (PMC10875040; doi:10.3389/fcvm.2024.1336609)
Supplement: Supplementary file 1 [file Datasheet1.pdf]

2018	Diagnosed with adenocarcinoma of left lung and developed brain metastases.
2018 to 2022	Treated with standard chemotherapy, targeted therapy and radiotherapy.
2022-12-28	Pembrolizumab was administrated.
2023-01-12	Displayed of shortness of breath, myalgias of limbs and fatigue.
01-15	New-onset CRBBB in local hospital; myalgias of limbs aggravated and diplopia appeared.
01-17	ECG showed ventricular tachycardia in our ED; **Syncope** occurred; Intravenous methylprednisolone 1 g/day and intravenous immunoglobulin (IVIG) 20 g/day were given for 3 days. Tocilizumab Injection of 640mg was used for 1 day.
01-18	Temporary pacemaker was implanted after treatment of atropine and isoproterenol.
01-20	Active fixation lead (Medtronic 5076-58) connected with an externalized generator (Medtronic Adapta ADSR01) was **placed**.
02-06	Symptoms improved; Myocardial enzymes continuously decreased.
02-20	Repeat ECG and 24h-holter showed **second-degree type I AVB.**
03-03	Invasive EPS demonstrated the heart block of supra-Hisian origin.
04-06	Corticosteroids were stopped.
04-13	7-days ECG monitoring showed 1:1 atrioventricular conduction at daytime.
04-25	**Remove** of active fixation lead and externalized generator.
05-18	ECG revealed **first-degree AVB** and CRBBB; complete recover of symtoms.
